# Targeting Antigens to Dendritic Cell Receptors for Vaccine Development

**DOI:** 10.1155/2013/869718

**Published:** 2013-10-08

**Authors:** Vasso Apostolopoulos, Theresia Thalhammer, Andreas G. Tzakos, Lily Stojanovska

**Affiliations:** ^1^VA Consulting Services, Melbourne, VIC, Australia; ^2^College of Health and Biomedicine, Victoria University, VIC, Australia; ^3^Institute of Pathophysiology and Allergy Research, Centre for Pathophysiology, Immunology and Infectiology, Medical University of Vienna, Vienna, Austria; ^4^Department of Chemistry, University of Ioannina, Ioannina, Greece

## Abstract

Dendritic cells (DCs) are highly specialized antigen presenting cells of the immune system which play a key role in regulating immune responses. Depending on the method of antigen delivery, DCs stimulate immune responses or induce tolerance. As a consequence of the dual function of DCs, DCs are studied in the context of immunotherapy for both cancer and autoimmune diseases. In vaccine development, a major aim is to induce strong, specific T-cell responses. This is achieved by targeting antigen to cell surface molecules on DCs that efficiently channel the antigen into endocytic compartments for loading onto MHC molecules and stimulation of T-cell responses. The most attractive cell surface receptors, expressed on DCs used as targets for antigen delivery for cancer and other diseases, are discussed.

## 1. Introduction

The most successful vaccines used to combat infectious disease are the live or live attenuated organisms as used in polio and small pox vaccines. However, with purified proteins or peptides, in most cases adjuvants or suitable danger signals are necessary in order to prime T-cell responses. In the last decade, dendritic cells (DCs), powerful antigen presenting cells, have surfaced as the most important cells, to target antigens for uptake, processing, and presentation to T cells [1]. DCs link the innate immune response to the adaptive immune response in that they bind pathogens and are able to stimulate T-cell responses against antigens. Targeting antigens to DC is therefore an appropriate method to stimulate effective immune responses. Targeting cell surface receptors on DCs represents a more direct and less laborious method and has been the subject of considerable recent investigation. Numerous receptors have been identified to be expressed on DCs, including mannose receptor (MR), DC-SIGN, scavenger receptor (SR), DEC-205, and toll-like receptors. Targeting of these receptors is becoming an efficient strategy of delivering antigens in DC-based anticancer immunotherapy. Furthermore, pattern recognition receptors (PRRs) are expressed by cells of the innate immune system which bind to pathogen associated molecular patterns (PAMPs) on pathogens. PRRs are also known as pathogen recognition receptors or primitive pattern recognition receptors as they evolved before other parts of the immune system, mainly before adaptive immunity. PAMPs bind mannose, lipopolysaccharide, fucose, peptidoglycans, lipoproteins and glucans. PRRs are classified into 2 groups: (i) endocytic PRRs, which phagocytose microorganisms, bind to carbohydrates, and include the mannose receptor (MR), glucan receptor, and scavenger receptor, and (ii) signaling PRRs which include the membrane bound toll-like receptors (TLR) and the cytoplasmic NOD-like receptors. The membrane bound receptors fall into 3 categories: (i) receptor kinases, (ii) TLR, and (iii) C-type lectin receptors. Targeting of these receptors is becoming an efficient strategy of delivering antigens in DC-based anticancer immunotherapy.

## 2. C-Type Lectin Receptors

**Figure 1:**
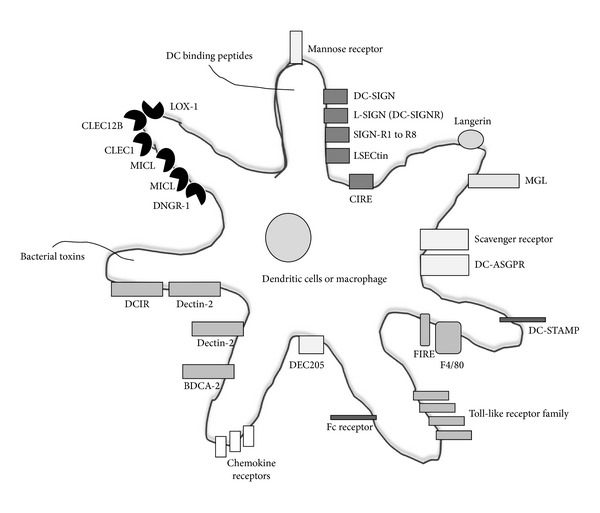
Schematic representation of dendritic cells expressing a number of different cell surface receptors which are targets for antigen targeting therapies.

Calcium-dependent (C-type) lectins consist of a large family of lectins which consist of carbohydrate recognition domains. The C-type lectin family includes the mannose receptor, mannose binding lectin, and ficolins and are active in immune-system functions such as pathogen recognition. In addition, dendritic cell C-type lectins, DC-SIGN, DC-SIGNR, DCAR, DCIR, Dectins, and DLEC are important in dendritic cell trafficking, formation of the immunological synapse, and inducing cellular and humoral immunity, bringing together both adaptive and innate immunity (Figure 1).

### 2.1. Group 1 C-Type Lectin Receptors: The Mannose Receptors

#### 2.1.1. Mannose Receptor

The mannose receptor (MR, CD206) is a C-type membrane lectin, carbohydrate (mannose, fucose, glucose, maltose, and GlcNAc) binding protein expressed by DCs and macrophages (Table 1 and Figure 1). MR binds to carbohydrates present on the cell walls of yeast, viruses, and bacteria, leading to endocytosis and phagocytosis [2]. Interestingly, human immunodeficiency virus (HIV) gp120 binds to MR on vaginal epithelial cells and induces the production of matrix metalloproteinases, facilitating transport of HIV across the vaginal epithelium [3]. In addition, HIV binds to the mannose receptor in sperm cells, suggesting that sperm cell-HIV interaction is an important source of infection [4]. The MR is part of the multilectin receptor family and provides a link between innate and adaptive immunity [5]. There are two types of MR in humans each encoded by its own gene, (i) mannose receptor C type 1 (MRC1) and (ii) mannose receptor C type 2 (MRC2).

The MR has been used as a target for vaccines, where DCs take up mannosylated proteins and utilize peptide epitopes for antigen presentation. The high expression of MR on DCs and macrophages suggests that the MR plays a key role in antigen recognition [6, 7]. The uptake of antigens by the MR allows processing and presentation via the MHC class I and II pathways [8–10], hence, suggesting MR a viable target for antigen delivery for vaccine development. Indeed, mannosylated peptides and proteins stimulate MHC class II specific T cells with 200 to 10,000-fold higher efficiency compared to peptides or proteins that are not mannosylated [10]. There is a 100-fold enhanced presentation of soluble antigens to T cells after being internalized by the MR on DCs, as compared to antigens internalized via fluid phase [9]. The MUC1 antigen conjugated to oxidized mannan (poly-mannose, comprising aldehydes) leads to rapid and 1,000 times more efficient MHC class I presentation to CD8+ T cells with a preferential T1 response, compared to MUC1 antigen conjugated to reduced mannan (no aldehydes) [8]. MUC1 antigen conjugated to reduced mannan results in class II presentation and a T2 immune response [8]. Both conjugate formulations, oxidized and reduced mannan, bind equally to the MR and are taken up into early endosomes [8]. MUC1-oxidized mannan rapidly escapes from the early endosomes into the cytosol for proteasomal processing and transport to the endoplasmic reticulum, Golgi apparatus, and MHC class I on the cell surface. By contrast, MUC1-reduced mannan remains in the early endosomes, to late endosomes, and to lysosomes, resulting in MHC class II presentation of antigens. Furthermore, both oxidized and reduced mannan stimulated bone marrow derived DCs, showed enhanced allogeneic T-cell proliferation, and enhanced OTI/OTII peptide specific T-cell responses *in vitro*. Mice injected with oxidized or reduced mannan induced a mature phenotype of lymph node and splenic DCs [11]. Oxidized and reduced mannan both stimulated upregulation of inflammatory cytokines interleukin-(IL-) 1beta and tumour necrosis factor-alpha; however, oxidized mannan stimulated IFN-gamma, IL-12p40 cytokines whereas reduced mannan stimulated IL-4, IL-10, and IL-13 [11]. Moreover, the activation of DCs was toll-like receptor-4 (TLR-4) dependent [11]. Thus, the mode of mannan conjugation to antigen is important as the differential immune responses result [12–18]. These studies provided the first demonstration that the MR aided antigens into both the MHC class I or II pathways depending on the chemical modification of mannan. In addition, *ex vivo* targeting of macrophages or DCs with oxidized mannan-MUC1 and reinjection into mice, induces strong CTL responses and protects against MUC1 tumor challenge [6, 19–21]. Humans are injected with oxidized mannan-MUC1 which induce cellular and humoral immune responses and protect against recurrence in breast cancer patients [21–24]. *Ex vivo* culture of human DC and pulsing with oxidized mannan-MUC1 and reinjection into patients with adenocarcinoma result in strong cellular immune responses and clinical responses [25]. Moreover, reduced mannan conjugated to myelin basic protein (MBP) 87–99 or 83–99 altered peptide ligands [26–28] (R^91^A^96^MBP_87-99_, A^91^A^96^MBP_87-99_, and Y^91^MBP_83-99_) divert Th1 IFN-gamma responses to Th2 IL-4 responses [29, 30]. Likewise, reduced mannan conjugated to cyclic A^91^A^96^MBP_87-99_ and A^91^MBP_83-99_ peptides significantly altered predominant Th1 responses to predominant Th2 responses [31–33]. Thus, mannan in its oxidized form has been shown to be effective as an anticancer vaccine, and mannan in its reduced form shows promise as a vaccine against autoimmune diseases such as multiple sclerosis.

DNA immunization is an attractive form of vaccination, which has shown promising results only in small animal models. Targeting the MR for DNA vaccines is a viable approach for the rational design of DNA vaccine strategies [34]. Mannosylated liposomes incorporating OVA DNA induced strong CTL responses in mice as compared to nonmannosylated complexes [35]. Complexation of oxidized or reduced mannan to OVA DNA via poly-l-lysine were able to stimulate strong cellular and humoral immune responses in mice [36, 37]. Using MUC1 DNA complexed to oxidized or reduced mannan was more immunogenic (T-cell responses, IFN-gamma secretion, low dose administration, and tumor protection) compared to MUC1 DNA alone [38]. In another approach, cationic amphiphiles containing mannose mimics, quinic acid, and shikimic acid headgroups are able to target the MR on DCs, leading to effective immune responses and tumor protection [39], suggesting that mannosylated DNA is an effective approach in generating immune responses.

Dendrimers are repetitive branched molecules which adopt a spherical 3-dimensional morphology. Dendrimers have 3 major parts, a core, an inner shell, and an outer shell, and attachment of compounds could be added in an attempt to develop novel immunotherapeutics. Mannosylated dendrimer OVA was shown to be taken up, processed, and presented by bone marrow derived DCs and Flt3-L DCs [40]. Mannosylated dendrimer OVA stimulated CD4+ and CD8+ T-cell responses and antibodies and protected mice against a OVA+ tumor challenge. Mannosylated dendrimer OVA induced DC maturation which was largely dependent on TLR-4 [41]. 

Mannan coated cationic liposomes (nanoparticles) incorporating HIV-1 DNA stimulate cytotoxic T lymphocytes (CTL), IFN-gamma, IgG2a, IgA, and delayed-type hypersensitivity responses [42]. The binding and uptake properties of mannan coated nanoparticles were 50% higher compared to the nonmannan coated nanoparticles, by MR+ cell line, J774E [43]. The binding and uptake were inhibited in the presence of free mannan, suggesting that the uptake was receptor dependent [43]. Anionic liposomes on the other hand, with the bilayer composition of phosphatidylcholine, cholesterol, phosphatidylglycerol, and phosphatidylserine do not bind to DCs. However, mannosylation of anionic liposomes increased their interaction to murine and human DCs, which could be blocked with free mannan [44]. Thus, the type of liposome is important in the development of effective vaccines, although mannan coating could overcome the pitfalls. Mannosylated liposomes incorporating ErbB2 CTL and T helper peptides and synthetic TLR2/1 or TLR2/6 agonists induced higher therapeutic efficacy compared to nonmannosylated liposomes [45]. In addition, mannosylated liposomes bind and are endocytozed by immature DCs; however, only nonspecific endocytosis is observed with nonmannosylated liposomes [46]. Liposomes conatining multibranched mannosylated lipids bind with higher affinity to the MR leading to effective uptake and endocytosis, compared to liposomes containing the monomannosylated analogs [46]. Furthermore, mannan coated poly(D, L-lactide-co-glycolic acid) and PLGA nanoparticles and enhanced CD4+ and CD8+ T-cell responses compared to nonmannan coated nanoparticles [47].

In addition, HER2 protein complexed to cholesteryl group-bearing mannan or pullulan polysaccharides generates CD8+ CTLs which reject HER2+ tumors in mice [48]. Furthermore, mannosylated chitosan microspheres (MCMs) incorporating Bordetella bronchiseptica antigen bound to the MR on murine macrophages (RAW264.7 cells) *in vitro* and induced strong IgA antibody responses *in vivo* [49]. However, mannose coated stealth microspheres, although bound to the MR, were not able to mature DCs *in vitro* [50]. 

Four lipid-core peptides were synthesized containing a sequence from the human papillomavirus type-16 (HPV-16) E7 protein (E744-62) and d-mannose. Immunization of mice with d-mannose-E7 peptide reduced or cleared tumors more effectively 37/40 compared to 21/30 in mice immunized with nonmannosylated peptides [51]. Numerous vaccines use keyhole limpet hemocyanin (KLH), to aid in antibody and T-cell responses. KLH activates and matures DCs by upregulating CD40, CD80, CD83, CD86, and MHC class II cell surface molecules and stimulating IL-12 and IL-10 cytokines [52]. The interaction of KLH to DCs was noted to be partially mediated by binding to the MR.

Cluster differentiation 1 (CD1) proteins, in particular, CD1b expressed on macrophages and DCs, present lipid antigens (including lipid mycolic acid and lipoarabinomannan) to T cells [53, 54]. The antigen presentation pathway for lipoarabinomannan has been characterized, and the MR is clearly responsible for uptake [55]. Lipoarabinomannan is endocytozed into early endosomes via the MR and from late endosomes is loaded onto CD1b molecules for T-cell presentation [55]. This study linked the MR to presentation of glycolipids via CD1 and suggests that the MR plays a major functional role in processing of carbohydrate antigens.

The melanoma associated antigen pmel17 fused to the heavy chain of an anti-MR antibody (B11-pmel17) and pulsed to DCs results in both MHC class I and class II presentation and CTL generation [56]. Likewise, human chorionic gonadotropin beta protein expressed by cancer cells, coupled to anti-MR antibody (B11-hCGbeta) generated MHC class I and class II T-cell responses and lysed hCGbeta+ cell lines [57]. T helper cells and CTL from cancer patients and healthy subjects were effectively primed with B11-hCGbeta pulsed DCs when a combination of TLR-ligands was used. It was evident that when TLR3 (poly I:C ligand) or TLR7/8 (resiquimod ligand, R-848) were used, concomitant signaling of DCs led to efficient antigen presentation by MR targeting [58]. Thus, MR and TLR together both contribute towards maturation and activation of DCs; in human clinical trials this was well tolerated with strong immune responses in cancer patients, and a phase II study is currently in progress [59, 60]. Similarly, NY-ESO-1, a cancer-testis Ag widely used in clinical cancer vaccine trials, was fused with either anti-MR or anti-DEC205 antibodies [61]. NY-ESO-1-antiMR antibody bound to the MR on DCs and NY-ESO-1-anti-DEC-205 on DCs, leading to stimulation of CD4+ and CD8+ T cells from peripheral blood mononuclear cells of cancer patients [61]. In contrast, nonantibody targeted NY-ESO-1 proteins only activated CD4+ T cells. Thus, targeting either the MR or DEC205 on DCs is a promising vaccination strategy to induce strong cellular immune responses.

In order to retain the characteristics of mannose rich carbohydrates and target the MR on DCs, antigens were expressed in yeast. Several recombinant ovalbumin (OVA) proteins were generated in *Pichia Pastoris* which naturally mannosylated OVA [62]. Mannosylated OVA induced enhanced antigen-specific CD4+ T-cell proliferation compared to non-mannosylated OVA, and, uptake was primarily due to mannose-specific C-type lectin receptors (MR and DC-SIGN) [63]. Further, stronger CTL responses and IFN-gamma, IL-2, IL-4, IL-5 cytokines were induced after vaccination in mice [64]. These studies demonstrate that yeast derived mannosylation of antigens enhances immunogenicity. Therapeutic strategies using tumor-specific immunoglobulin (idiotype, Id) for lymphomas are promising. Id proteins are usually produced via tumor-myeloma hybridomas or recombinant methods in mammalian, bacteria, or insect cells. Using insect cells, the Id produced contain mannose residues which have enhanced immunostimulatory properties (activation of DCs, CD8+ T-cell stimulation, and eradication of lymphomas), compared to Id proteins made in mammalian cells [65]. However, anti-lymphoma antibodies generated by Id insect cell compared to mammalian cells were similar. Thus, insect derived antigens are far more immunostimulatory compared to mammalian derived antigens, primarily due to the expression of mannose which binds to the MR.

Humans with suppressed T cells have high prevalence of *Cryptococcosis*. Soluble Cryptococcus neoformans mannoproteins (MP) are promising vaccine candidates due to their ability to induces delayed-type hypersensitivity and Th1 cytokines. MP binds to the MR and results in CD4+ T-cell stimulation and induce protective responses against *C. neoformans* and *Candida albicans*. The uptake of MP by DCs can be inhibited either by competitive blockade of the MR or by removal of carbohydrate residues critical for recognition [66]. Further, MPs increased the expression of CD40, CD83, CD86, MHC class I and II cell surface moleules, and IL-12 leading to the maturation and activation of DCs [67]. It was clear that the mannose groups on MP provided the immunogenicity of cryptococcal MP and this finding supports vaccination strategies that target the MR.

It is clear that antigen mannosylation is an effective approach to potentiate antigen immunogenicity, due to the enhanced antigen uptake and presentation by DCs and macrophages.

### 2.2. Group 2 C-Type Lectin Receptors: Asialoglycoprotein Receptor Family

#### 2.2.1. DC-SIGN

Dendritic cell-specific intercellular adhesion molecule-3-grabbing nonintegrin, (DC-SIGN ) also known as CD209, Clec4L, is a C-type membrane lectins abundantly expressed on immature DCs, macrophages, endothelial vascular cells, atherosclerotic plaques, and lymphatic vessels, but not on plasmacytoid DCs (Table 1 and Figure 1). Like the MR, DC-SIGN recognizes carbohydrates including mannose, fucose, N-acetylgalactosamine, and N-acetylgiucosamine residues on pathogens mediating endocytosis, thus activating and tailoring the adaptive immune response against pathogens. DC-SIGN also binds yeast derived mannan and Lewis blood group antigens and sialylation or sulfation of Le^x^ completely abrogated binding to DC-SIGN [68]. DC-SIGN contributes to HIV pathogenesis. HIV-1 gp120, binds to DC-SIGN on monocyte derived DCs more than 80% with residual binding to CD4, as opposed to HIV-1 only binding to CD4 on blood DCs [69]. After binding to DC-SIGN on DCs, HIV-1 is transported by DCs into lymphoid tissues and consequently facilitates HIV-1 infection of target CD4+ T cells [70, 71]. DC-SIGN also has high affinity binding for ebola virus, hepatitis C virus, dengue virus, respiratory syncytial virus, measles virus, *Mycobacterium tuberculosis*, *Leishmania amastigote*, *Helicobacter pylori*, *Leishmania mexicana*, *Schistosoma mansoni*, *Porphyromonas gingivalis*, *Neisseria gonorrhoeae*, and *Candida albicans*, transmitting infection (virus, bacteria, and yeast) to susceptible cells and, inducing Th1 Th2 T cell responses [72–77]. Recently, it was shown that DC-SIGN is the receptor for the major house dust mite (Der p1) and dog allergens (Can f1) [78]. There is no binding of DC-SIGN with *E. coli*, *Klebsiella pneumoniae*, *Pseudomonas aeruginosa*, and *Staphylococcus aureus *[68]. DC-SIGN was identified through its high affinity interaction with ICAM-3 which facilitates DC interactions with T cells and contributes to the regulation of primary immune responses [70, 71]. DC-SIGN also interacts with ICAM-2 which is responsible for DC migration [79]. In view of these findings, DC-SIGN has implications for antigen targeting and stimulation of T-cell responses and has been studied as a potential receptor for vaccine targeting. 

In order to understand the molecular basis of internalization of ligands by DC-SIGN, the putative internalization motif within the cytoplasmic tail was modified resulting in reduced internalization after exposure to antigen [80]. DC-SIGN ligand complexes are internalized by DCs into late endosomes, early lysosomes, and are processed and presented to CD4+ T cells [80]. Further, anti-DC-SIGN monoclonal antibodies are internalized up to 1,000-fold more efficiently compared to control monoclonal antibody and found in intracellular vesicles, indicating that targeting DC-SIGN targets the MHC class II pathway [81]. Anti-DC-SIGN monoclonal antibody conjugated to KLH was rapidly internalized into the lysosomal compartment of DCs and induced up to 100-fold increase stimulation of T cells compared to KLH alone pulsed DCs [82]. In addition, anti-DC-SIGN antibody-KLH-targeted DCs induced proliferation of naive T cells which recognized KLH T-cell epitopes presented by MHC class I and II molecules [82] and inhibited tumor cell growth in mice [83]. These studies use an anti-DC-SIGN monoclonal antibody that binds to the carbohydrate recognition domain. Recently, an anti-DC-SIGN monoclonal antibody which binds to the neck region of DC-SIGN was rapidly internalized into early endosomes by DCs by a clathrin-independent mechanism, unlike anti-DC-SIGN antibodies which target the carbohydrate recognition domain are internalized into late endosomes, via a clathrin dependent mechanism [84]. Further, enhanced (up to 1,000-fold) T-cell stimulation resulted using the antineck region DEC205 antibody [84]. Hence, targeting different regions of DEC205 results in distinct internalization modes, and shows potential for targeted vaccination strategies.

Hamster bone marrow derived DCs, expressing high levels of DEC205 and DC-SIGN, pulsed with tumor lysates of hamster pancreatic cells and injected into tumor bearing hamsters reduced tumor growth significantly [85], further demonstrating that targeting DC-SIGN or DEC205 receptors may be useful for the development of effective vaccines. Liposomes containing calcein are rapidly taken up by immature and mature myeloid DCs [86], and nanoparticles but not microparticles deliver antigen to human DCs via DC-SIGN *in vitro *[87], further demonstrating DC-SIGN as a targeted receptor for vaccine design.

The melanoma antigen, Melan-A/Mart-1 (peptide 16–40, containing the CD8+ HLA-A2 restricted T-cell epitope, amino acids 26–35), was coupled to either Manalpha-6 Man or lactoside, or a Lewis oligosaccharide [88]. The glycoconjugates containing Lewis oligosaccaride bound with high affinity to DC-SIGN were taken up by DCs into acidic vesicles and presented by MHC class I and stimulated CD8+ T-cell responses [88]. However, glycoconjugates containing lactoside were not taken up by DCs. Modification of the melanoma antigen, gp100, with glycans (high mannose) interacted specifically with DCs and induced enhanced CD4+ T-cell responses [89]. Further, Le^x^ oligosaccharides conjugated to OVA targeted DC-SIGN on DCs effectively and stimulated CTL and IFN-gamma secretion (but not IL-10) by T cells and required 300-fold lower dose to immunize compared to OVA immunization alone [90]. Using human DC-SIGN transgenic DCs, Le^x^-OVA was efficiently endocytozed and enhanced OT-I CD8+ and OT-II CD4+ T-cell stimulation resulted, compared to OVA alone [91]. The heparanase tumor antigen is not able to elicit an immune response; however, conjugation of heparanase to Le^x^ was able to stimulate IFN-gamma cytokine secretion by T cells, CTL responses and delay the growth of established tumors in mice [92]. Liposomes modified to express Le^x^ and LeB increased binding and internalization by human DCs which was further enhanced, up to 100-fold, and stimulated both CD4+ and CD8+ T-cell responses, in the presence of lipopolysaccharide, compared to nonmodified liposomes. In addition, modified liposome-Le^x^LeB encapsulating the melanoma antigen MART-1 in the presence of lipopolysaccharide also enhanced CD8+ T-cell clone activation *in vitro *[93]. Polyamidoamine dendrimers comprising LeB antigen are taken into lysosomes, and dendrimers containing at least 16–32 glycan units are necessary for antigen presentation and cytokine production [94]. Thus, complexes using Le oligosaccharides to target DC-SIGN represent a novel method for vaccination against tumor antigens. Likewise, lentivirus vectors modified with Sindbis virus envelope proteins, when linked to OVA, are taken up by murine bone marrow derived DCs and stimulate OT-I and OT-II T cells, CTL *in vivo* and protects mice against the challenge of OVA expressing tumor cells [95]. The binding of the modified lentivirus vectors with Sindbis virus envelope proteins to DC-SIGN is mannose dependent. Further modification of the vector to include 1-deoxymannojirimycin and to inhibit mannosidases (an enzyme that removes mannose structures during glycosylation) resulted in enhanced antibody responses [96]. These studies demonstrate that glycoconjugates could be designed to target DC-SIGN for developing tumor vaccines. The use of glycans to target DC-SIGN has advantages over anti-DC-SIGN monoclonal antibodies, as they reduce the risk of side effects and their generation relies purely in organic chemistry approaches. However, a recent study demonstrated that receptor-specific antibodies are more effective at inducing immune responses than carbohydrates (glycans) for DC-targeted vaccination strategies [97].

*L-SIGN or DC-SIGNR*. L-SIGN or DC-SIGNR (also known as CD299, CD209L, and Clec4M) is a type-II transmembrane C-type lectin receptor homologous to DC-SIGN (77% amino acid sequence homology), highly expressed on liver sinusoidal cells, endothelial vascular cells, and in the lymph nodes, but not on DCs, in contrast to DC-SIGN (Table 1 and Figure 1). Like DC-SIGN, L-SIGN has a high affinity binding to ICAM-3, HIV, simian immunodeficiency virus, Ebola virus, hepatitis C virus and respiratory syncytial virus [72, 73, 75]. L-SIGN also binds with HIV gp120-binding protein and Man9GlcNAc2 oligosaccharide, and binding is enhanced up to 25-fold with Man9GlcNAc2 di-saccharide [98]. Antibodies against L-SIGN, are taken up by human liver sinusoidal endothelial cells and a cross-reactive antibody to L-SIGN/DC-SIGN conjugated to tetanus toxoid induced T-cell responses against tetanus toxoid. Thus, targeting L-SIGN shows promise for the development of targeted vaccines [99].

A further 8-mouse homologs to human DC-SIGN have been documented: SIGN-related gene 1 (SIGN-R1), SIGN-R2, SIGN-R3, SIGN-R4, SIGN-R5, SIGN-R6, SIGN-R7, SIGN-R8 [100]. The carbohydrate specificity of SIGN-R1 (CD209b) and SIGN-R3 is similar to DC-SIGN, in that they bind mannose- and fucose-containing ligands and interact with Lewis blood antigens; however, SIGN-R1 and SIGN-R3 also interact with sialylated Le^x^, a ligand for selectins [101, 102]. SIGN-R1 also binds to zymosan, to the capsular polysaccharide of *S. pneumoniae*, and with low affinity to dextran and is highly expressed by macrophages [101, 103–105]. Bovine serum antigen (BSA) consisting, 51 mannoside residues (Man(51)-BSA) binds to SIGN-R1 on lamina propria DCs in the gastrointestinal tract and induces IL-10 cytokine secretion by DCs, but not IL-6 and IL-12p70 [106]. *In vitro* and *in vivo*, Man(51)-BSA stimulates CD4+ type 1 regulatory T-like cells (Tr-1) but not CD4+CD25+Foxp3+ regulatory T cells, suggesting that SIGN-R1 induces tolerance to antigens [106].

*LSECtin*. LSECtin (liver and lymph node sinusoidal endothelial cell C-type lectin, Clec4G) is a type-II transmembrane C-type lectin protein, similar to the related proteins DC-SIGN and L-SIGN and is expressed in liver, lymph node cells, and sinusoidal endothelial cells but not monocyte derived DCs (Table 1). LSECtin binds to N-acetyl-glucosamine and fucose but does not bind to galactose and may function *in vivo* as a lectin receptor [107]. LSECtin is coexpressed with DC-SIGNR and CD23 and binds to ebola virus, filovirus glycoproteins, lymphocytic choriomeningitis virus, and, to the S-protein of SARS coronavirus but does not interact with HIV-1 and hepatitis C [108]; although a study suggested that LSECtin binds to hepatitis C virus, the interaction was in association DC-SIGNR with [109]. Ligands binding to LSECtin are not inhibited by mannan but by EDTA suggesting that the LSECtin does not bind to mannose [108]. Recently, LSECtin was shown to bind with CD44 [110]. Another study, regarding the expression of LSECtin demonstrated LSECtin, to be expressed on human peripheral blood, thymic DCs, monocyte-derived macrophages and DCs [111], and to human Kupffer cells [112]. Antibody or ligand-mediated engagement of LSECtin activates rapid internalization of LSECtin [111] indicating that LSECtin may be a suitable receptor for targeting antigens in the development of vaccination regimes. Further work is required to determine the viability of LSECtin to be an appropriate target for immunotherapy studies.

*CIRE*. CIRE (C-type lectin immune receptor, CD209) is a murine type 2 membrane protein which belongs to the C-type lectin receptors and is preferentially expressed by immature CD8− splenic DCs (CD8−CD4+ and CD8−CD4−), on some CD4+ DCs, and on plasmacytoid pre-DCs, with no expression on CD8+ DCs, macrophages, or monocytes (Table 1 and Figure 1) [113]. CIRE that has 57% identity with DC-SIGN is the murine homolog to human DC-SIGN and both bind mannose residues [114]. However, CIRE is downregulated after activation, and incubation with cytokines IL-4 and iL-13 does not enhance expression of CIRE, even though DC-SIGN is enhanced, suggesting differences in gene regulation between the two receptors [113]. CIRE consists of 238 amino acids, and its extracellular domain contains a C-type lectin domain; it is the ligand for ICAM-3 and is a receptor for HIV binding facilitating trans-infection of T cells. Importantly, CIRE does not bind with ebola virus glycoprotein, *Leishmania mexicana*, cytomegalovirus, and lentivirus, which are defined ligands for DC-SIGN [113]. The lack of interaction is due to defect in multimerization of CIRE which is thought to be necessary for pathogen recognition by DC-SIGN [115], suggesting that CIRE and DC-SIGN have functional differences.

Polyanhydride nanoparticles covalently linked to d-mannose and lactose increased the cell surface expression of CD40, CD86, MHC class II, CIRE, and MR on bone marrow derived DCs, compared to nonmodified nanoparticles, although both nanoparticles were similarly internalized [116]. In addition, polyanhydride nanoparticles linked to galactose and d-mannose, increased the cell surface expression (CD40, CD86, MHC class I and II, CIRE, MR and macrphage galactose lectin) and proinflammatory cytokines (IL-1beta, IL-6, and TNF-alpha) on alveolar macrophages [117]. Likewise, polyanhydride microparticles linked to (1,6-bis(p-carboxyphenoxy)hexane (CPH) and sebacic acid) or (1,8-bis(p-carboxyphenoxy)-3,6-dioxaoctane and CPH) were rapidly phagocytosed within 2 hours by bone marrow derived DCs and increased cell surface expression of CD40, CD86, MHC class II and CIRE, and cytokines IL-12p40 and IL-6 [118]. Conjugation of the microparticles to OVA stimulated CD8+ OT-I and CD4+ OT-II T cells [118]. Blocking MR and CIRE inhibited the upregulation of cell surface molecules on DCs, suggesting that CIRE and MR engage together for DC activation [116]. CIRE shows promise as an appropriate target for antigen delivery for improved vaccine development.

#### 2.2.2. Langerin

Langerin (CD207, Clec4K) is a type-II transmembrane cell surface receptor highly expressed on Langerhans cells, CD103+ DCs, and splenic CD8+ DCs (Table 1). Langerin is a C-type lectin which highly binds to mannose residues which are internalized by DCs into Birbeck granules (where Langerin is localized) where there is access to the nonclassical antigen processing and presentation pathway.

A comparative study between murine DC-SIGN, SIGN-R1, SIGN-R3, and Langerin demonstrated functional differences amongst the different C-type lectins, despite similarities in the carbohydrate recognition domains. Murine DC-SIGN did not bind dextran, OVA, zymosan, or heat-killed *Candida albicans*, but SIGN-R1, SIGN-R3, and Langerin showed distinct carbohydrate recognition [119]. Only SIGN-R1 bound to *Escherichia coli* and *Salmonella typhimurium* (Gram-negative bacteria), and neither murine DC-SIGN, SIGN-R1, SIGN-R3 nor Langerin bound to *Staphylococcus aureus* (Gram-positive bacteria) [119]. In addition, SIGN-R1 (but not the other lectin receptors) distinctively bound to zymosan [119]. Langerhans cells (a subset of DCs) are divided into two groups: (i) Langerhans cells that express Langerin and (ii) epidermal Langerhans cells that go to lymph nodes, which function and develop independently [120]. Anti-Langerin monoclonal antibody targeted to Langerin was efficiently endocytozed by Langerhans cells *in vitro* [121] and *in vivo* [122], suggesting further studies in immunizations through the skin for DC-based vaccination therapies. Indeed, anti-Langerin monoclonal antibody conjugated to HIV gag-p24 induced Th1 and CD8+ T-cell responses in mice [123]. Interestingly, anti-DEC-205 monoclonal antibody was recently shown to be taken up by Langerin-positive DCs [124], suggesting there is cross-talk between DEC-205 and Langerin receptors. Further, a noncovalent fusion between anti-Langerin monoclonal antibody and HA1 influenza hemagglutinin elicited antigen-specific T-cell and antibody responses *in vitro* and *in vivo* [125].

#### 2.2.3. MGL

MGL (human macrophage galactose- and N-acetylgalactosamine-specific C-type lectin) is the classical asialoglycoprotein receptor (Figure 1). MGL is highly expressed on macrophages and immature DCs, whose ligand specificity differs from DC-SIGN and L-SIGN, in that it binds to galactose and N-acetylgalactosamine leading to Th2 skewed immunity [126, 127]. In addition, MGL binds the strongest to serine, threonine O-linked glycosylated Tn antigen, a well-known human carcinoma-associated epitope, and not to sialylated Tn antigen [128, 129]. Moreover, hMGL binds to the group of filoviruses and to gonorrhea (via lipooligosaccharides) leading to altered DC cytokine secretion profiles and stimulation of CD4+ Th responses (Table 1) [77, 126, 127].

MUC1 peptide (3 tandem repeats, 60 amino acids enzymatically glycosylated with GalNAc) or short MUC1 or MUC2 peptides containing Tn bound to immature DCs and the MUC1-Tn glycopeptide localized within the MHC class I and class II compartments [130]. MUC1 glycopeptides linked to anti-MGL antibody led to upregulation of human DC cell surface molecules and enhanced CD8+ T stimulation *in vitro* [131]. In mice, MGL+ CD103- dermal DCs bound to glycosylated Tn antigen* in vivo*, stimulating MHC class II CD4+ T-cell responses. Intradermal immunization with Tn-glycopeptides generates antibodies and Th2 cytokine secretion by CD4+ T cells [132]. Recently, a mimic of galactose/N-acetylgalactosamine stimulated blood monocytes and myeloid derived DCs [133], suggesting that glycosylated mimetics could be used to target antigens to MGL expressing DCs. These results demonstrate that the targeting of MGL receptor expressed on murine and human DCs stimulates T-cell and antibody responses, and this approach could be used to design novel anticancer vaccines.

#### 2.2.4. Dectin-1 Subfamily

Dectin-1 (dendritic cell-associated C-type lectin-1, DCAL-1, Clec7A) or beta-glucan receptor is a C-type lectin receptor which is part of the NK gene complex in the Dectin-1 cluster (Table 1 and Figure 1) [134]. It was originally characterized to be DC specific (hence its name), but it is now known to be also expressed on myeloid DCs, CD8−CD4− DCs, dermal DCs, monocytes, macrophages, neutrophils, microglia, T-cell subsets, B cells, mast cells, eosinophils, and monocytes [134–136]. Dectin-1 is a receptor for beta-glucan recognizing beta1,3 and beta1,6-linked glucans on yeast, mycobacterial, and plant cell walls and plays a role in innate immune responses [137, 138]. Zymosan, a beta-glucan and mannan-rich ligand binds to Dectin-1 [139], and Dectin-1 interacts with the tetraspanin molecule CD37. Dectin-1 binds to *Saccharomyces*, *Candida*, *Pneumocystis*, *Coccidiodes*, *Penicillium*, and *Aspergillus*, but not *Cryptococcus *fungal species, leading to activation of Dectin-1+ cells and elimination of fungal pathogens by activating inflammatory responses, such as TNF-alpha, CDCL1, IL-1beta, GM-CSF, and IL-6, by the presence of an ITAM in its cytoplasmic tail [135]. In fact, Dectin-1 knockout mice are highly susceptible to pathogenic infections due to inflammatory defects and reduced fungal killing [140]. Furthermore, Dectin-1 binds to bacteria resulting in TNF-alpha, IL-6, RANTES, G-CSF, and IL-12 secretion [141]. The stimulation of inflammatory and Th1 cytokines leads to the proposal of Dectin-1 targeting of soluble antigens by appropriate ligands to stimulate cellular immunity.

Anti-Dectin-1 and anti-Dectin-2 monoclonal antibodies conjugated to OVA [142, 143] and induced significant expansion of T cells in the draining lymph nodes of mice and IFN-gamma secretion by T cells [142, 143]. Purified beta1,3-d-glucan from *Saccharomyces cerevisiae* cell wall, free from mannan and other proteins, binds to Dectin-1 receptor on DCs. Beta1,3-d-glucan conjugated to OVA matures bone marrow derived DCs was rapidly phagocytosed and stimulated >100-fold more efficiently CD8+ OT-I and CD4+ OT-II T cells, compared to OVA alone [144]. Immunization of mice with beta1,3-d-glucan stimulated IgG2c antibodies, CD4+ T cells, IFN-gamma, and Th17 biased responses [144]. Thus, robust stimulation of humoral and cellular immune responses results following immunization with vaccine candidates that target Dectin-1 receptor.

*DNGR-1*. DNGR-1 (NK lectin group receptor-1, Clec9A) is a group V C-type lectin-like type II membrane protein located close to Dectin-1 encoded within the NK gene complex. DNGR-1 is expressed on murine CD8+ DCs not on CD4+ DCs, on CD11c+ DCs but not by CD11c− cells (B cells, T cells, NK cells, NKT cells, macrophages, and granulocytes), on plasmacytoid DCs, and on a small subset of human blood DCs (BDCA-3+ DCs) and monocytes (CD14+CD16−) and induces proinflammatory cytokines [145, 146]. DNGR-1 is also not expressed by interstitial DCs, in skin epidermis, and on GM-CSF derived bone marrow DCs but highly expressed on Flt3 ligand bone marrow derived CD8+ DCs (CD11b^low^CD24^hi^B220−) [146]. Anti-DNGR-1 monoclonal antibody covalently conjugated to CD8+ peptide from OVA, induced OT-I CD8+ T-cell proliferation and IFN-gamma secretion *in vivo*, and only CD8+ DCs and not plasmacytoid DCs were involved in the presentation of the peptide to CD8+ T cells [146]. In the presence of anti-CD40, CTLs are primed *in vivo* and prevent OVA+ expressing tumor cell growth [146]. Injection of anti-DNGR-1 monoclonal antibody-OVA conjugate into mice was endocytozed by CD8+ DCs, presented antigen to CD4+ T cells, and played a major role in the differentiation of CD4+ T cells into Foxp3+ regulatory T cells [147]. The addition of the adjuvant poly I:C enhanced IL-12 mediated immunity, whereas the adjuvant curdlan primed Th17 cells [147]. In addition, vaccinia virus infected dying cells are endocytozed by DNGR-1 on DCs and mediate cross-priming of antivaccinia virus infected cell CD8+ T-cell responses; loss of DNGR-1 impairs CD8+ CTL responses [148, 149]. Thus, DNGR-1 regulates cross-presentation of viral antigens and could be further assessed as a target for vaccination protocols. Furthermore, a single injection of anti-Clec9A monoclonal antibody induced striking antibody and CD4+ T cells responses in the absence of adjuvant or danger signals in mice and in TLR knockout mice [150, 151]. Targeting antigens to Clec9A shows promise to enhance vaccine efficiency; indeed, anti-Clec9A monoclonal antibody conjugated to HIV gag-p24 induced strong Th1 and CD8+ T-cell responses in mice [123]. DNGR-1/Clec9A could prove useful for developing immunotherapy protocols for cancer and other diseases.

*MICL*. MICL (myeloid inhibitory C-type lectin-like receptor, Clec12A) is homologous to Dectin-1 and is part of the Dectin-1 cluster [152]. Numerous other groups identified this receptor and named it C-type lectin-like molecule-1 (CLL-1), DC associated C-type lectin 2 (DCAL-2), and killer cell lectin-like receptor 1 (KLRL1) [153–155]. MICL is expressed on granulocytes, monocytes, macrophages, B cells, CD8+ T cells in peripheral blood, and DCs (Table 1) [156], and, contains a tyrosine based inhibitory motif in its cytoplasmic tail, similar to lectin-like receptor for oxidized density lipoprotein-1 (LOX-1) and Dectin-1, and can inhibit cellular activation. Hence, MICL is a negative regulator of granulocytes and monocytes [152]. MICL has a range of functions including cell adhesion, cell-cell signaling, turnover of glycoproteins, and in inflammation and in immune responses.

*CLEC2*. CLEC2 (also known as Clec1B), a C-type lectin-like receptor 2, is expressed on NK cells, DCs, monocytes, granulocytes, platelets, megakaryocytes, and liver sinusoidal endothelial cells (Table 1) [157]. CLEC2 is a platelet activation receptor for the endogenous ligand, podoplanin (a mucin-like sialoglycoprotein) expressed on a number of cells including lymphatic endothelial cells and implicated in cancer cell metastasis [158]. CLEC2 on platelets binds to HIV-1 and facilitates HIV-1 spread to other immune cells. The binding of HIV-1 to platelets via CLEC2 is highly dependent on DC-SIGN, suggesting that the two coexist [159]. In addition, the snake venom rhodocytin binds to CLEC2 on platelets and activates cell signaling [160]. Not much is known about CLEC2 and stimulation of immune responses, but its expression on DCs and its colocalization with DC-SIGN suggest it may have immune stimulatory effects.

*CLEC12B*. CLEC12B (macrophage antigen H) is part of the NK gene complex/Dectin-1 cluster of C-type lectin receptors, highly expressed on macrophages, monocytes, and DCs and contains immunoinhibitory sequences in its cytoplasmic tail [161, 162]. There not much known regarding CLEC12B and its function on DCs and macrophages. It is possible that CLEC12B could be used as a receptor to target antigens for immunotherapy studies for diseases, including cancer; however, this is still to be determined.

*LOX-1.* LOX-1 (lectin-like receptor for oxidized density lipoprotein-1, Clec8A) is part of the Dectin-1 cluster of C-type lectin receptors. LOX-1 is also considered to be a member of the scavenger receptor family. LOX-1 is expressed on endothelial cells, smooth muscle cells, platelets, fibroblasts, and macrophages and binds to Gram-positive and gram-negative bacteria, oxidized-LDL modified lipoproteins, phospholipids, apoptotic cells, C-reactive protein, and heat shock protein (HSP)-70 [163]. LOX-1 does not contain the classical signaling motifs in its cytoplasmic tail but is involved in endocytosis, phagocytosis, cytokine production, and in the production of reactive oxygen species [164, 165]. As a consequence of the binding of LOX-1 to HSP-70, DC-mediated antigen cross-presentation results [166]. An anti-LOX-1 monoclonal antibody which inhibits the binding of HSP-70 to DCs also inhibits HSP-70 induced cross-presentation of antigens. Anti-LOX-1 monoclonal antibody linked to OVA protein specifically stimulated CD4+ OVA T-cell hybridoma *in vitro* as measured by IL-2 production [166]. Injection of anti-LOX-1-OVA conjugated into mice prevented the growth of OVA expressing tumor cells [166]. Hence, targeting LOX-1 is a promising target for cancer immunotherapy studies.

#### 2.2.5. DC Immunoreceptor (DCIR) Subfamily

*DCIR*. DCIR (DC immunoreceptor) is a C-type lectin receptor, with tyrosine based immune-inhibitory functions, Clec4A). DCIR is primarily expressed on plasmacytoid DCs (pDCs), on immature and mature monocyte-derived DCs, on monocytes, macrophages, and B cells, and after maturation of pDCs, DCIR is reduced (Table 1). Binding to TLR9 on pDCs induces IFN-alpha, which is inhibited by DCIR activations whilst costimulatory molecules are not affected [167]. DCIR has a range of functions including cell adhesion, cell-cell signaling, turnover of glycoproteins, and in inflammation and in immune responses. Targeting DCIR is rapidly internalized into clathrin pits and processed and presented to T cells [167]. An anti-DCIR monoclonal antibody is rapidly internalized by human monocyte derived DCs into endolysosomal vesicles and does not unregulate TLR4 nor TLR8 mediated upregulation of costimulatory molecules, CD80 and CD86, but does inhibit TLR8 mediated IL-12 and TNF-alpha production [168]. Thus, targeting DCIR activates T cells but also inhibits TLR8-induced (IL-12 and TNF-alpha production) and TLR9-induced (IFN-alpha production), which may be applied in vaccine development for disease prevention and treatment. Targeting antigens to DCIR were evaluated for their potential to stimulate CD8+ T-cell responses. Anti-DCIR monoclonal antibody linked to influenza matrix protein, melanoma antigen MART-1, or to HIV gag antigens resulted in expansion of CD8+ T cells *in vitro* [169] and stimulation of Th1 and CD8+ T cells *in vivo* [123]. The addition of TLR-7/8 agonists enhanced T expansion of primed CD8+ T cells and induced the production of IFN-gamma and TNF-alpha and reduced the levels of Th2 cytokines [169]. It is clear that, antigen targeting via the DCIR activates specific CD8+ T-cell immune responses.

*Dectin-2*. Dectin-2 (or DCAL-2, Clec6A) or beta-glucan receptor is a C-type lectin receptor expressed on DCs, macrophages, neutrophils, and monocytes (Table 1) [170]. Dectin-2 is a receptor for beta-glucan recognizing beta1,3 and beta1,6-linked glucans on yeast, mycobacterial, and plant cell walls and plays a role in innate immune responses [137, 138]. Anti-Dectin-2 monoclonal antibody conjugated to antigen stimulate, CD8+ T cells in mice [142]. In addition, a lentivector using the mouse Dectin-2 gene promoter, was taken up by bone marrow derived DCs, Langerhans cells, and dermal DCs *in vitro* [171]. The Dectin-2 lentivector encoding the human melanoma antigen, NY-ESO-1, stimulated CD4+ and CD8+ T cells in mice [171]. Thus, Dectin-2 expressed on DCs is a potential targeting protein for vaccinations.

*BDCA-2.* Blood DC antigen 2 (BDCA-2, Clec4C) is a type II C-type lectin expressed on human blood DCs, which has 57% homology with its murine homolog Dectin-2. Anti-BDCA-2 monoclonal antibody is rapidly internalized by plasmacytoid DCs and presented to T cells and suppresses the induction of IFN-alpha/beta cytokine secretion [172].

## 3. DEC205 

DEC-205 (CD205 or lymphocyte antigen Ly 75) is a type-I integral membrane protein homologous to the macrophage MR family of C-type lectins, which binds carbohydrates and mediates endocytosis (Figure 1) [173]. DEC-205 is primarily expressed on DCs and thymic epithelial cells. DEC205 mediates a number of different biological functions, such as binding and internalization of ligands for processing and presentation by DCs (Table 2). Although the ligands which bind to DEC205 are not clear, following ligand binding, DEC-205 is rapidly internalized by means of coated pits and vesicles and is delivered to multivesicular endosomal compartments that resemble the MHC class II-containing vesicles implicated in antigen presentation. Due to the endocytic properties of DEC205, it is a promising receptor for antigen delivery for vaccines and targeted immunotherapies [174]. Upon DC maturation, DEC205 is upregulated, unlike other members of the macrophage MR family.

In an attempt to design vaccines that target DEC205, the cytosolic tail of DEC-205 was fused to the external domain of the CD16 Fc gamma receptor and was studied in stable L cell transfectants [175]. The DEC-205 tail recycled CD16 through MHC II-positive late endosomal/lysosomal vacuoles and also mediated a 100-fold increase in antigen presentation to CD4+ T cells. An anti-DEC-205 monoclonal antibody conjugated to OVA was shown to stimulate OVA-specific CD4+ and CD8+ T cells by CD11+ lymph node DCs, but not by CD11c− DCs [176]. Injection of anti-DEC-205-OVA conjugate in mice was taken up by draining lymph node DCs and stimulated CD8+ T (OT-I) cells 400 times more efficiently compared to OVA alone; this response was further enhanced *in vivo* (as measured by IL-2, IFN-gamma, CTL, and tumor protection), with the addition of anti-CD40 antibody (a DC maturation stimulus) [176]. Further, anti-DEC-205 antibody-OVA intradermally injected in mice was rapidly taken up by Langerhans cells and stimulated both CD4+ and CD8+ T-cell responses [122]. Langerin positive skin DCs play a major role in transport of anti-DEC-205-OVA complex, although Langerin negative dermal DCs and CD8+ DCs were responsible for the T-cell stimulation [124]. Hence, there is cross-talk between DC subsets. 

Conjugation of the anti-DEC-205 monoclonal antibody to the melanoma antigen tyrosinase-related protein TRP-2, induced CD4+ and CD8+ T-cell responses which protected mice against B16 tumor cell growth and slowed growth of established B16 tumors [177]. In addition, anti-DEC205 monoclonal antibody linked to survivin (a survival protein overexpressed on carcinoma cells) together with anti-CD40 and poly I:C stimulated surviving-specific CD4+ T-cell responses (IFN-gamma, TNF-alpha, IL-2 secretion), lytic MHC class II+ T cells but not CD8+ T cells. Depletion of CD25+foxp3+ cell prior to immunization led to further enhanced immune responses [178]. Interestingly, HER2/neu protein expressed on breast cancer cells was genetically engineered into anti-DEC205 monoclonal antibody, and in combination with poly I:C and CD40 antibody, elicited robust CD4+ and CD8+ T-cell responses and antibody responses which protected mice against Her2+ breast tumor challenge [179]. Further, HIV p24 gag protein conjugated to anti-DEC205 monoclonal antibody, or HIV gag p24-single chain DEC-205 Fv DNA vaccines, was taken up by DCs and stimulated proliferation and IFN-gamma secretion by CD8+ T cells that had been isolated from HIV-infected donors [180, 181]. Similarly, in mice, immunization led to Th1 (IFN-gamma, IL-2), CD4+ and CD8+ T-cell responses, and 10-fold higher antibody levels [123, 181–183]. Likewise, priming with the DNA vaccine and boosting with adenoviral vector (comprising anti-DEC205 monoclonal antibody conjugated to OVA or HIV-1 gag together with anti-CD40) induced strong CD8+ T-cell responses; no enhanced effect was seen with the addition of TLR-9 ligand CpG and TLR-3 ligand poly I:C or CD40 ligand [184]. Recombinant Newcastle disease virus vaccine vector (rNDV) on its own induces IFN-alpha and IFN-beta production and DC maturation. Immunization with rNDV encoding anti-DEC205 and HIV-1 gag antigen enhanced CD8+ gag specific T-cell responses and increased the number of CD4+ and CD8+ T cells in the spleen compared to rNDV encoding gag antigen alone [185]. Furthermore, mice were protected against challenge of recombinant vaccinia virus expressing HIV gag protein [185]. Conjugation of anti-NLDC-145 monoclonal antibody (monoclonal antibody against murine DEC205) to a model antigen stimulated both antibody and T-cell responses in animal models [186]. Conversely, using a self antigen, proteolipid protein (PLP_139-151_) conjugated to anti-DEC205 monoclonal antibody tolerized T cells *in vivo* and reduced the secretion of IL-17 by CD4+ T cells and *in vitro* CD4+Vbeta6+ T-cell receptor T cells specific for PLP_139-151_ became anergic [187]. Hence, targeting self-antigens to DEC-205 induces tolerance. It is clear that, targeting DCs using DEC-205 directed antibody-antigen conjugates represents a novel method of inducing tolerance to self-antigens and antitumor immunity *in vivo*. 

## 4. Scavenger Receptor

The scavenger receptors (SRs) are a group of receptors that recognize modified low density lipoprotein (LDL) by oxidation (oxLDL) or acetylation (acLDL) (Figure 1). Scavenger receptor was given its name based on its “scavenging” function. SR is primarily present on macrophages internalize endotoxins, oxLDL, and other negatively charged proteins. SR, are grouped into classes A, B, and C according to their structural features. (i) Scavenger receptor class A (SR-A1, SR-A2) is mainly expressed on macrophages as a trimer and has 6 domains (cytosol, transmembrane, spacer, alpha-helical coiled-coil, collagen-like, and cystein-rich domains) (Table 2). Members include SCARA1 (MSR1), SCARA2 (MARCO), SCARA3, SCARA4 (COLEC12), and SCARA5. (ii) Class B (SR-B1) has 2 transmembrane regions and are identified as as ocLDL receptors. Members include SCARB1, SCARB2, and SCARB3 (CD36). (iii) Class C has a transmembrane region in which the N-terminus is located extracellularly. There are other receptors that have been reported to bind to oxLDL which include CD68 and its murine homolog macrosialin, mucins, and LOX-1.

Despite the scavenging functions of SR, SRs have been shown to endocytoze antigens and present antigens to MHC class I and II and stimulate effective CD4+ and CD8+ T-cell responses. Using 200 nm particles coated with oligonucleotide polyguanylic acid (SR-targeting agent) showed specific binding to SR, and particles were localized in intracellular vesicles and processing via the endocytotic pathway [188]. An early example demonstrating immune responses generation was with maleylated OVA which bound to SR, enhancing its presentation and stimulation of CTLs by macrophages and B cells [189]. Maleylated diphtheria toxoid was also more immunogenic than nonmaleylated diphtheria toxoid, generating enhanced antibody and T-cell proliferative responses [190]. Likewise, in chickens, immunization with maleylated bovine serum albumin yielded Th1 immune response via antibodies. In addition, high levels of IFN-gamma mRNA were detected in splenocytes compared to nonmaleylated bovine serum antigen that stimulated Th2 immune responses [191]. Tropomyosin from shrimp causes allergic responses in some individuals inducing a dominant Th2 cytokine profile and IgE antibody responses. Modifying tropomyosin to maleylated tropomyosin, diverted responses from IL-4 Th2 dominant proallergic phenotype to an IFN-gamma Th1 antiallergic phenotype. Thus, modification of proteins to target the SR on macrophages elicits Th1 IFN-gamma responses [192]. SRs recognize malondialdehyde and acetaldehyde adducted proteins [193] and when linked to hen egg lysozyme protein, stable adducts (oxidative products) are formed. Immunization in mice results in strong T-cell proliferative and antibody responses [193]. MARCO, a SR class A family member expressed on murine macrophages and human monocyte-derived DCs, plays an influential role in mediating immune responses. Anti-MARCO antibody linked to tumor lysate-pulsed DCs enhance, tumor-reactive IFN-gamma producing T cells and reduced tumor growth in mice [194]. These studies demonstrate the implications of targeting antigens to MARCO and other SRs for use in human clinical DC vaccine trials.

### 4.1. DC-ASGPR

DC-asialoglycoprotein receptor (DC-ASGPR) is a lectin-like scavenger receptor. It is expressed on monocyte derived DCs (CD14+CD34+), on tonsillar interstitial-type DCs and granulocytes, but not on T cells, B cells, NK cells, monocytes, Langerhans cells, and CD1a derived DCs (Table 2) [195]. Anti-DC-ASGPR monoclonal antibody is rapidly internalized into early endosomes, indicating that DC-ASGPR is involved in antigen capture and processing [195]. Targeting DC-ASGPR induces a suppressive CD4+ T-cell response that secretes IL-10 *in vitro* and *in vivo *[196]. Hence, targeting antigens to DC-ASGPR induces antigen specific IL-10-producing suppressive T cells, and DC-ASGPR could be utilized to induce a suppressive immunotherapeutic effect to self- or non-self-antigens.

## 5. F4/80 Receptor

F4/80 is restricted to macrophages, and for over 40 years F4/80 has been used to identify and characterize macrophages in tissues and its functional role in macrophage biology [197]. F4/80 is the murine homolog of the epidermal growth factor-like module containing mucin-like hormone receptor-1 protein encoded by the EMR1 gene. F4/80 although highly expressed on macrophages does not play a role in macrophage development (Table 2 and Figure 1). However, F4/80 receptor was found to be necessary for the induction of CD8+ T regulatory cells responsible for peripheral immune tolerance [197]. No ligands to F4/80 are known, and much work is still required to understand the role of F4/80 in the immune response and could be a novel antigen targeting receptor.

### 5.1. FIRE

FIRE is an F4/80-like receptor expressed specifically on CD8−CD4+ and CD8−CD4− immature DCs and weakly on monocytes and macrophages (Table 2) [198]. Rat anti-FIRE (6F12) and rat anti-CIRE (5H10) antibodies (targeting the FIRE and CIRE receptors on CD8− DCs) were injected into mice, and anti-rat Ig titres were measured and compared to control rat antibody [198]. Anti-FIRE and anti-CIRE IgG1 antibody responses were 100–1,000-fold greater to non-targeted control rat antibody. The magnitude of the responses was equivalent to that seen when CpG was included as an adjuvant [198]. Conversely targeting the DEC205 receptor, expressed on CD8+ DCs with rat anti-DEC-205 antibody (NLDC-145), did not induce humoral immune responses unless CpG was added [198]. This study demonstrated the differences in the ability of CD8+ and CD8− DC subsets to stimulate immune responses *in vivo*.

## 6. DC-STAMP

DC-specific transmembrane protein (DC-STAMP) contains 7 transmembrane regions and has no sequence homology with other multimembrane cell surface receptors and has an intracellular C-terminus. DC-STAMP resides in the endoplasmic reticulum, where it interacts with LUMAN (also known as CREB3 or LZIP) of immature DCs and upon stimulation DC-STAMP translocates to the Golgi apparatus and is expressed on the cell surface upon maturation [199]. DC-STAMP is specifically expressed by DC, on activated but not resting blood DCs, and not in a panel of other leukocytes or nonhematopoietic cells (Table 2) [200]. DC-STAMP lentiviral vector-OVA in mice tolerize OT-I CD8+ and OT-II CD4+ T-cell responses, leading to elimination and functional inactivation of CD4 and CD8 T cells in peripheral organs and in the thymus [201]. Binuclear and multinuclear DCs express low levels of MHC class II and IL-12p70 with high levels of IL-10 which suppress T-cell proliferative responses [202]. Blocking of DC-STAMP decreased the number of binuclear cells, suggesting that the DC-STAMP is responsible for the immunosuppresive effects of binucleated DCs [202]. Thus, targeting antigens to DC-STAMP tolerize antigen specific T-cell responses *in vivo*. Conversely, using DC-STAMP promoter driven construct linked to OVA, resulted in strong OVA-specific CD4+ and CD8+ T-cell responses *in vitro* and *in vivo* and protected mice against OVA+ tumor challenge [203]. Thus, DC-STAMP shows promise as a target for cancer vaccine antigen targeting approach.

## 7. Fc Receptor

Fc receptors (FcR) for immunoglobulins link humoral and cellular immune responses [204]. They also link the innate immune response to the adaptive immune response by binding to pathogens and immune complexes and stimulating T cells. There is a different FcR for each class of immunoglobulin Fc*α*lphaR (IgA), Fc*ε*psilonR (IgE), Fc*γ*ammaR (IgG), and Fc*α*lpha/*μ*egaR (IgA and IgM). There are 4 types of Fc*γ*ammaR: Fc*γ*ammaRI (CD64), Fc*γ*ammaRII (CD32), Fc*γ*ammaRIII (CD16), and Fc*γ*ammaRIV. It is becoming evident that antibody-antigen complexes present antigen more efficiently than antigen alone via the Fc*γ*ammaR. OVA antigen complexed with anti-OVA antibody injected into mice is presented 10 times more efficiently to T cells compared to OVA alone [205]. An interesting study demonstrated that *γ*amma-chain knockout mice which lack Fc*γ*ammaRI/Fc*γ*ammaRIII/Fc*γ*ammaRIV induced similar CD8+ T-cell responses in mice compared to the wild-type mice. However, CD8+ T-cell proliferative responses were reduced in Fc*γ*ammaRI/Fc*γ*ammaRII/Fc*γ*ammaRIII knockout mice compared to wild type mice, suggesting that all FcR other than Fc*γ*ammaRIV take up immune complexes and stimulate CD8+ T-cell responses [205]. In a comparative study between FcR and MR targeting of prostate serum antigen (PSA), PSA antigen/anti PSA antibody complex induced both CD4+ and CD8+ T-cell responses however, mannose-PSA stimulated only CD4+ T cells [206]. However, given that the antigen is mannosylated in the appropriate form, CD8+ T cells could be generated, as seen with oxidized versus reduced mannan-MUC1 conjugates (Table 2) [6, 8, 12, 13, 21].

### 7.1. Fc*γ*ammaRIII (CD16)

Fc*γ*ammaRIII is also known as CD16. Conjugation of tetanus toxoid 14 amino acid peptide or a hepatitis C virus peptide to anti-CD16 antibody activated CD4+ T-cell clones 500 times more effectively compared to peptide alone [207]. Hence, Fc*γ*ammaRIII has properties of antigen uptake, processing, and presentation to T cells for effective immune response generation.

### 7.2. Fc*α*lphaRI (CD89)

Fc*α*lphaRI is expressed on myeloid cells, interstitial-type DCs, CD34+ DCs, and monocyte derived DCs [208]. Fc*α*lphaR1 binds to *Porphyromonas gingivalis*, *Bordetella pertussis,* and *Candida albicans* stimulating efficient immune responses for their elimination [209–213]. Cross-linking of Fc*α*lphaRI induced internalization of receptor and activation of DCs; however, there was very minimal antigen presentation [214, 215]. Therefore, it is unlikely that targeting antigen to human Fc*α*lphaRI will result in generating increased immune responses. 

### 7.3. Fc*ε*psilonRII (CD23)

Fc*ε*psilonRII (CD23) is a type 2 transmembrane C-type lectin that binds with low affinity to IgE. CD23 also interacts with CD21, CD11b, and CD11c. Unlike other Fc receptors, CD23 is a C-type lectin. Its main function is in allergic responses, and it is expressed on activated B cells, activated macrophages, eosinophils, platelets, and follicular DCs. CD23 is noncovalently associated with DC-SIGN and MHC class II on the surface of human B cells. Following endocytosis of anti-CD23 antibodies, CD23 is lost from the cells; however, endocytosis anti-MHC class II antibody leads to recycling of HLA-DR-CD23 complex to the cell surface, consistent with the recycling of MHC class II in antigen presentation; CD23 is internalized into cytoplasmic organelles that resembled the compartments for peptide loading (MHC class II vesicles) [216]. This may lead to peptide presentation, and the return of CD23 with MHC class II to the cell surface may aid in the stabilization of B-cell-T-cell interactions, leading to T-cell responses [216]. It is apparent that human and murine B cells take up IgE-antigen complexes via CD23 and present antigenic peptides via MHC class II stimulating CD4+ T cells. TNP-(trinitrophenyl-) specific IgE linked to BSA or OVA and injected into mice results in 100-fold enhanced IgG antibody responses as compared to either IgE or BSA or OVA injected alone; the enhanced antibody effects are completely dependent on CD23 [217, 218]. In addition, the coexpression of CD23 with DC-SIGN further suggests that antigen presentation and stimulation of antigens is possible between the cross-talk of these two receptors. Hence, targeting CD23 is a novel vaccine strategy for stimulating CD4+ T-cell immune responses.

## 8. Conclusions

A promising strategy to improve the immunogenicity of antigens is “antigen targeting.” DCs are unique in their ability to present antigen to naive T cells and, hence, play a major role in initiating immune responses. Characterization of DC receptors aid in the understanding of the mechanism underlying their potent antigen presenting capacity. A major challenge for vaccine design is targeting antigens to DCs *in vivo*, facilitating cross-presentation, and conditioning the microenvironment for Th1- and Th2-type immune responses. We have analysed numerous DC cell surface receptors, which function in inducing cellular responses and individually each shows promise as targets for vaccine design against cancer. More recently there has been an upsurge of information regarding toll-like receptor (TLR) targeting and stimulation of DCs via TLR. It is clear that in mice, use of TLR ligands to activate DCs stimulates effective cellular immune responses and activation of DCs. However, no substantial TLR-targeting vaccine trials have been completed in humans and it remains to be determined whether TLR targeted approach will result in significant benefits in humans as those seen in mice. Furthermore, targeting antigens to chemokine receptors [1] on DCs (CCR1, CCR2, CXCR4, CCR5, CCR6, and CXCR1) generates enhanced immune responses *in vitro* and *in vivo.* Furthermore, bacterial toxins, DC binding peptides and internalization peptide (Int) also target antigens to DCs; however, the targeting does not involve receptor targeting. It is clear that receptor targeting of antigens is a promising new approach for cancer immunotherapy studies.

## Figures and Tables

**Table 1:** Summary of dendritic cell receptors targeted for vaccine development: C-type lectin receptors.

Receptor	Designation	Function
1. Group 1 C-type lectin receptors		
1.1. Mannose receptor	CD206	Expressed on macrophages and DCs. Binds to mannan, mannose, fucose, glucose, maltose, GlcNAc, lipoarabinomannan, cell wall of yeast, viruses, and bacteria leading to phagocytosis/endocytosis. Used to target protein, peptides, DNA, dendrimers, liposomes, and anti-MR antibodies for vaccine development with Th1, Th2, CTL, and Ab responses induced. Targeting antigens to MR using mannan has been used in human clinical trials.

2. Group 2 C-type lectin receptors		
2.1. Dendritic cell-specific intercellular adhesion molecule-3-grabbing nonintegrin (DC-SIGN)	CD209 Clec4L	Expressed on immature DCs, macrophages endothelial vascular cells, atherosclerotic plaques, and lymphatic vessels, not on placmacytoid DCs. Binds to mannan, mannose, fucose, GlcNAc, GalNAc, yeast, lewis blood group antigens Le^x^, HIV-1 gp120, ebola virus, hepatitis C virus, dengue virus, respiratory syncytial virus, measles virus, *Mycobacterium tuberculosis, Leishmania amastigote, Helicobacter pylori, Leishmania mexicana, Schistosoma mansoni, Porphyromonas gingivalis, Neisseria gonorrhoeae*, *Candida albicans*, house dust mite (Der p1), and dog allergens (Can f1). Interacts with ICAM-3 and ICAM-2. Targeting DC-SIGN using antigen linked to anti-DC-SIGN antibodies, Manalpha-6 Man, lactoside, and Lewis oligosaccharide, stimulates T-cell and/or antibody responses, and has been studied as a potential receptor for vaccine targeting. Eight murine homologues identified, SIGN-R1 (CD209b) to SIGN-R8.
2.1.1. L-SIGN or DC-SIGNR	CD299 CD209L Clec4M	Expressed on liver sinusoidal cells, lymph nodes, and endothelial vascular cells, but not on DCs. Binds to HIV gp120, Man9GlcNAc2, HIV, simian immunodeficiency virus, ebola virus, hepatitis C virus, and respiratory syncytial virus. Targeting L-SIGN with anti-L-SIGN antibodies induces T-cell responses. Targeting L-SIGN shows promise for the development of targeted vaccines.
2.1.2. Liver and lymph node sinusoidal cell type lectin (LSECtin)	Clec4G	Expressed in liver, lymph nodes, sinusoidal endothelial cells, DCs, and Kupffer cells. Binds to N-acetyl-glucosamine, fucose, ebola virus, filovirus glycoproteins, lymphocytic choriomeningitis virus, S-protein of SARS coronavirus, and to CD44, but not to mannose, HIV, and hepatitis C. Coexpressed with DC-SIGNR and CD23. Antibody or ligand-mediated engagement of LSECtin activates rapid internalization, indicating that LSECtin may be a suitable receptor for targeting antiges in the development of vaccination regimes.
2.1.3. C-type lectin immune receptor (CIRE) (murine homologue of DC-SIGN)	CD209	Expressed by immature CD8− splenic DCs (CD8−CD4+ and CD8−CD4−), on some CD4+ DCs, plasmacytoid pre-DCs, and not by, CD8+ DCs, macrophages, or monocytes. It is a ligand for ICAM-3 and binds to HIV. Polyanhydride nanoparticles covalently linked to dimannose and lactose matures DCs and are internalized by DCs. CIRE shows promise as an appropriate target for antigen delivery for improved vaccine development.
2.2. Langerin	CD207 Clec4K	Expressed on Langerhans cells, CD103+ DCs, and splenic CD8+ DCs. Binds to mannose and internalizes mannose residues into Birbeck granules, where Langerin is expressed. Anti-Langerin antibody targeting antigens to Langerin is endocytozed *in vitro* and *in vivo* and induces Th1 and antibody responses.
2.3. MGL (human macrophage galactose- and N-acetylgalactosamine-specific C-type lectin)		Expressed on macrophages, immature DCs galactose, GalNAc, Tn antigen, filoviruses, and gonorrhea. GalNAc modified peptides to target MGL receptor expressed on murine and human DCs, which stimulates T-cell and antibody responses, and this approach could be used to design novel anticancer vaccines.
2.4. Dectin-1 or beta-glucan receptor (DC-associated C-type lectin-1)	DCAL-1 Clec7A	Expressed on myeloid DCs, CD8−CD8− DCs, dermal DCs, monocytes, macrophages, neutrophils, T cells, B cells, mast cells, eosinophils, and monocytes. Binds to beta-glucan on yeast, mycobacteria, plant cell walls, *Saccharomyces, Candida, Pneumocystis, Coccidioides, Penicillium,* and *Aspergillus*, but not *Cryptococcus* fungal species, and interacts with CD37. Anti-Dectin-1 and anti-Dectin-2 antibodies linked to proteins stimulate CD8+ and CD4+ T cells, and immunization with beta-glycan modified proteins induces CD4+ and Th17 bias responses.
2.4.1. DNGR-1 (NK lectin group receptor-1)	Clec9A	Expressed on murine CD8+ DCs not on CD4+ DCs, on CD11c+ DCs but not by CD11c− cells (B cells, T cells, NK cells, NKT cells, macrophages, and granulocytes), on plasmacytoid DCs, and on human blood DCsBDCA-3+ DCs) and monocytes (CD14+CD16−). Highly expressed on Flt3 ligand bone marrow derived CD8+ DCs. Target for immune response induction.
2.4.2. Myeloid inhibitory C-type lectin receptor (MICL)	Clec12A	Homologous to Dectin-1 and part of Dectin-1 cluster. Also termed as CLL-1, DCAL-2, and KLRL1. Expressed on granulocytes, monocytes, macrophages, B cells, CD8+ T cells in peripheral blood, and DCs.
2.4.3. C-type lectin-like receptor 2 (CLEC2)	Clec1B	Expressed on NK cells, monocytes, granulocytes, platelets, megakaryocytes, and liver sinusoidal epithelial cells. Binds to HIV-1 and facilitates HIV-1 spread to other cells and binds to snake venom rhodocytin. Not much is known regarding stimulating immune responses; however, colocalization with DC-SIGN suggests that it may have an immune stimulatory effect.
2.4.4. CLEC12B (macrophage antigen H)	Clec21B	Part of the NK gene complex/dectin-1 cluster of C-type lectin receptors. Expressed on macrophages, monocytes, and DCs. Not much is known regarding its function.
2.4.5. LOX-1 (Lectin-like receptor for oxidized density lipoprotein-1)	Clec8A	Part of the dectin-1 cluster of C-type lectin receptors and scavenger receptor family. Expressed on endothelial cells, smooth muscle cells, platelets, fibroblasts, and macrophages. Binds to Gram-positive and Gram-negative bacteria, oxidized LDL modified lipoproteins, phospholipids, apoptotic cells, C-reactive protein, and heat shock protein (HSP)-70. Targeting LOX-1 induces immune responses and is a promising target for cancer immunotherapy.
2.5. DC immunoreceptor subfamily		
2.5.1. DC immunoreceptor (DCIR)	Clec4A	Expressed on plasmacytoid DCs, immature and mature monocyte-derived DCs monocytes, macrophages, and B cells. Binds to TLR9. Targeting DCIR stimulates immune responses especially CD8+ T cells.
2.5.2. Dectin-2 (or beta-glucan receptor)	DCAL-2 Clec6A	Expressed on DCs, macrophages neutrophils, and monocytes. Binds to beta1,3 and beta1,6-linked glucans on yeast, mycobacteria, and plant cell walls. Targeting dectin-2 stimulates immune responses in mice.
2.5.3. Blood DC antigen (BDCA-2)	Clec4C	Expressed on human blood DCs. Targeting BDCA-2 suppresses IFN-alpha/beta cytokine secretion.

**Table 2:** Summary of dendritic cell receptors targeted for vaccine development: other receptors.

Receptor	Designation	Function
3. Type-1 integral membrane proteins		
3.1. DEC205	CD205	Homologous to the mannose receptor.
	Ly 75	Expressed on DCs and thymic epithelial cells. Targeting DEC205 induces an array of immune responses.

4. Scavenger receptors		
4.1. Scavenger receptor		Expressed on macrophages. Bind to modified low density lipoproteins (LDL) by oxidation (oxLDL) or acetylation (acLDL). Bind to CD68, macrosialin, mucins, and LOX-1. Targeting of scavenger receptors induces immune responses in mice.
4.1.1. Scavenger receptor class A	SR-A1	Expressed on macrophages as a trimer.
	SR-A2	Members include SCARA1 (MSR1), SCARA2 (MARCO), SCARA3, SCARA4 (COLEC12), and SCARA5.
4.1.2. Scavenger receptor class B	SR-B1	Consists of 2 transmembrane units. Members include SCARB1, SCARB2, and SCARB3 (CD36).
4.1.3. Scavenger receptor class C	SR-B1	Consists of a transmembrane region in which the N-terminus is located extracellularly.
4.2. DC-asialoglycoprotein receptor (DC-ASGPR)		A lectin-like scavenger receptor. Expressed on monocyte derived DCs (CD14+CD34+), tonsillar interstitial-type DCs, and granulocytes. Targeting DC-ASGPR induces suppressive responses.

5. F4/80 receptor		Expression restricted to macrophages. Murine homolog of the epidermal growth factor-like module containing mucin-like hormone receptor-1 protein encoded by the EMR1 gene.
5.1. FIRE		Expressed on CD8−CD4+ and CD8−CD4− immature DCs, and weakly on monocytes and macrophages. Targeting FIRE stimulates immune responses in mice.

6. DC-specific transmembrane protein (DC-STAMP)		Expressed on DCs and activated blood DCs. Targeting DC-STAMP results in immunosuppressive responses in some studies and in other studies stimulates strong cellular responses.

7. FcR		Links humoral and cellular immune (Fc Receptor) responses, links innate and adaptive immune responses by binding pathogens and immune complexes, and stimulates T cells. Targeting FcR is a novel vaccine strategy for stimulating immune responses.
